# The pathogenesis and treatment of posterior reversible encephalopathy syndrome after neuromyelitis optica spectrum disorder: a case report and literature review

**DOI:** 10.1186/s12883-022-02985-8

**Published:** 2022-12-20

**Authors:** Bo Yang, Lei Guo, Xu Yang, Nengwei Yu

**Affiliations:** 1 Department of Center for Psychosomatic Medicine, Sichuan Provincial Center for Mental Health,Sichuan Provincial People’s Hospital, University of Electronic Science and Technology of China, Chengdu, China; 2 Department of Neurosurgery, Sichuan Provincial People’s Hospital, University of Electronic Science and Technology of China, Chengdu, China; 3 Department of Encephalopathy, Traditional Chinese Medicine Hospital of Leshan, Leshan, China; 4 Department of Neurology, Sichuan Provincial People’s Hospital, University of Electronic Science and Technology of China, Chengdu, China

**Keywords:** Neuromyelitis optica spectrum disorder, Posterior reversible encephalopathy syndrome, Aquaporin-4, Immunotherapy

## Abstract

**Background:**

Posterior reversible encephalopathy syndrome (PRES) is a rare disease characterized by reversible subcortical vasogenic brain edema. Neuromyelitis optica spectrum disorder (NMOSD) is a frequent neurological autoimmune disease that is rarely reported to complicate PRES.

**Case presentation:**

Here, we report a case of neuromyelitis optica (NMO) concurrent with PRES. A 50-year-old woman presented with severe impairment of her health visual acuity, with significantly worsening of the motor weakness in both lower limbs during methylprednisolone therapy after her diagnosis of NMO. MRI showed new-onset brain edematous lesions of the bilateral frontal, occipital, and parietal lobes. PRES was considered. Her vision impairment and weakness of the extremities were alleviated after antihypertensive treatment and dehydration. The edema lesions detected by MRI also completely disappeared.

**Conclusions:**

We reviewed 14 cases of NMO with PRES and concluded that the etiology of NMOSD concurrent PRES may be multifactorial, involving pathogenic IgGs against aquaporin-4 (AQP-4) and immunotherapy treatment. Different underlying pathogeneses require different treatment approaches.

## Background

Posterior reversible encephalopathy syndrome (PRES) is an acute neurological disease with high reversibility and a good clinical prognosis. It is currently believed to be closely related to factors such as cytotoxic drug use, hypertension, renal dysfunction, and the presence of autoimmune diseases [1]. Neuromyelitis optica spectrum disorder (NMOSD) is an autoimmune disorder of the central nervous system in which autoantibodies characteristically attack both the optic nerve and spinal cord [2]. Until now few cases of NMOSD concurrent with PRES have been reported, and the pathogenesis remains unclear. Here, we report such a case of PRES following an NMOSD diagnosis and review previous case reports.

## Case presentation

The 50-year-old woman in question was diagnosed with optic neuritis in March 2021, presenting with progressive impairment of visual acuity isolated to the right eye, and despite opportune methylpred-nisolone treatment, still had deteriorating vision as determined by a finger-counting test. In September 2021, both fluctuant bilateral limb numbness and unstable gait successively occurred within 1 week, with accompanying palpitations and nausea. In February 2022, the above symptoms were aggravated and spasms were occurring in the left leg. During the course of the disease, the face of the woman was always swelling and her weight was reduced by 7 kg. She was then admitted to our hospital on April 18, 2022. Upon examinations conducted at admission, her blood pressure was 129/91 mmHg; she retained normal left vision and pupillary light reflex, but right pupil light reflex decreased; neurological examinations showed grade 4 muscle strength of the limbs, hypermyotonia of the limb and neck muscles, limb and gait ataxia, and bilateral hyperreflexia with the Babinski sign.

**Fig. 1:**
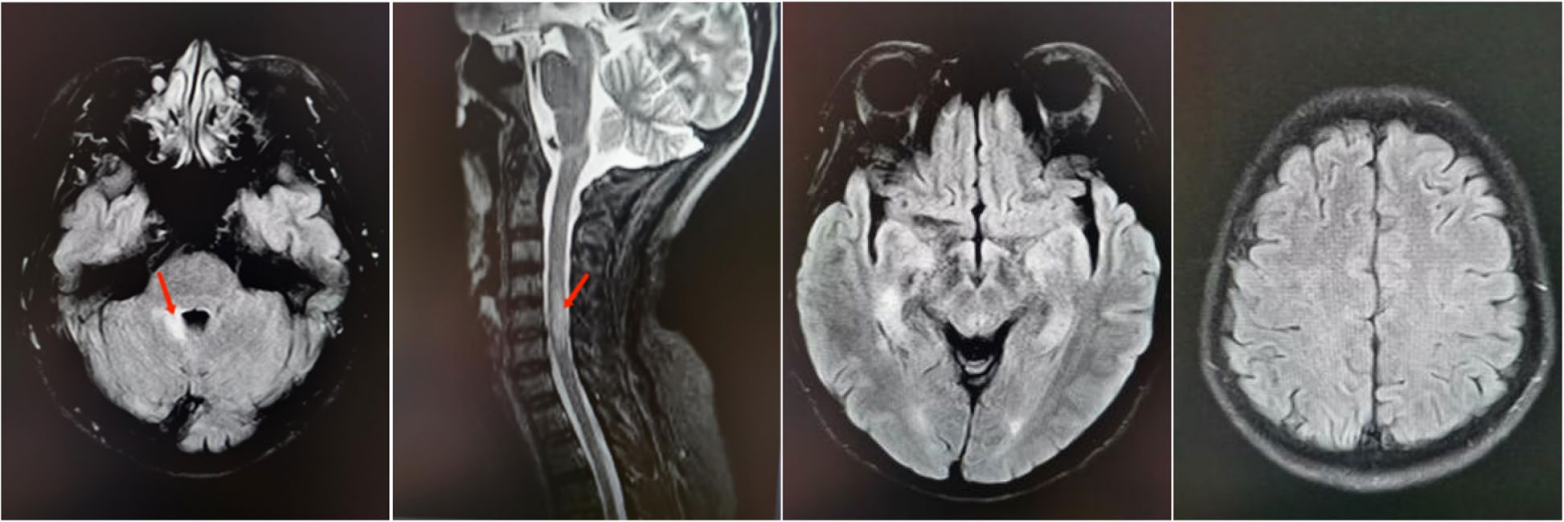
MRI shows T2 hyperintense at the dorsal pons near right surface of the fourth ventricle and spinal cord of the C4-C6 vertebrae,no lesions in bilateral occipital, parietal, and frontal lobes

Excluding the abnormally high values for triglycerides, total cholesterol, and low-density lipoprotein, the patient’s blood tests were otherwise normal (including routine, biochemistry, coagulation function tests, anti-neutrophil cytoplasmic antibodies (ANCA), anti-nuclear antibody (ANA), and thyroid function). Abdominal ultrasonography found suspicious uterine fibroids and gallbladder polyps. Electrocardiogram and chest CT were normal. MRI showed T2 hyperintensity of the dorsal pons near the right surface of the fourth ventricle and spinal cord of the C4-C6 vertebrae (Fig. 1). The cerebrospinal fluid (CSF) showed a raised WBC count (8*106/L) and elevated protein (0.528 g/L). Serum and CSF antibodies to AQP4 were positive. Accordingly, the patient was diagnosed with neuromyelitis optica spectrum disorder (NMOSD).

**Fig. 2:**
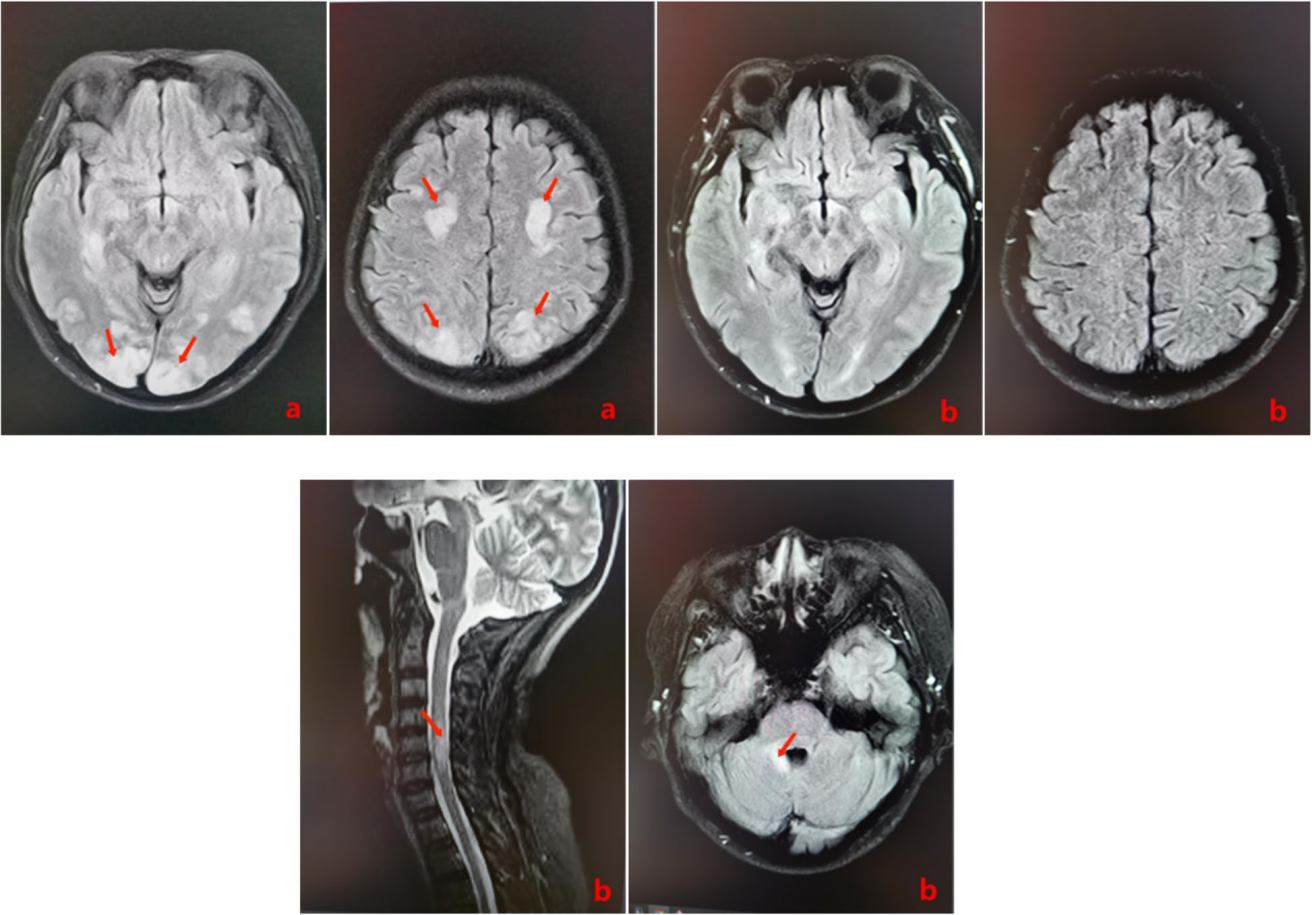
**a** MRI shows that bilateral frontal, occipital,and parietal new-onset brain gyrus edematous lesions (April 27, 2022). **b** MRI shows the disappearance of bilateral frontal, occipital,and parietal new-onset brain gyrus edematous lesions and residual lesions of the focal lesions at the dorsal pons near right surface of the fourth ventricle and spinal cord of the C4-C6 vertebrae

She was started on IV methylprednisolone (1 g per day) on April 22, 2022, and a combination of baclofen and clonazepam were used to improve the spasms. During the Initial treatment, her facial swelling exacerbated and blood pressure values indicated hypertension (155/65–175/75 mmHg). On the morning of 27 April 2022, her left vision had markedly deteriorated based on the finger-counting test, and her lower limb muscle strength was reduced. Immediate MRI reexamination indicated new-onset brain edematous lesions of the bilateral frontal, occipital, and parietal lobes (Fig. 2a). No abnormal hematological rechecks were found. Considering diagnosis of PRES, dehydration and hypertension-controlling measures were applied. The patient’s left vision and muscle strength improved on the next day and recovered to the status of admission after 5 days. With subsequent MRI examination on May 10, 2022, the edematous lesions had disappeared (Fig. 2b).

## Discussion and conclusion

We searched for case reports on NMOSD with PRES published before July 2022 using the databases of Pubmed, Embase, Sinomed, CNKI, and Wanfang. The search terms were as follows: posterior reversible encephalopathy syndrome; posterior reversible leukoencephalopathy syndrome; optic neuritis; neuromyelitis optica and neuromyelitis optica spectrum disorders. We found 14 cases of NMOSD with PRES across 10 papers. The clinical details are summarized in Table 1.

**Table 1:** Epidemiological, clinical and imaging datas of previous cases of NMOSD with PRES

Year	Age/sex	Diagnosis	Symptoms of PRES	NMO duration (y)	NMO attack preceding PRES (interval to PRES,d)	Blood pressure at time of PRES,(mmHg)	Inducing factor (interval to PRES,d)	Treatment for PRES	PRES lesion locations	PRES lesion symmetry	Time(d) and degree of MRI lesions resolution	References
2009	56/F	rLETM	Coma,Nystagmus, Diplopia	2	Yes(9)	80/60	PLEX/1d	No	F,P,O,C,Corpus callosum, Thalamus	Yes	23d,Complete	[3]
2009	46/F	rLETM	Delirium, Cortical blindness	6	Yes(13)	146/86	IVIg/7d	PLEX	F,P,O,C	Yes	10d,Complete	[3]
2009	57/F	NMO	Sleepiness, Delirium,Coma	24	No	220/140	No	IVMP,AH	F,P,O,C,Corpus callosum	Yes	4d,Partial	[3]
2009	48/F	NMO	Sleepiness,Diplopia	8	No	120/74	IVMP/1d	No	P,O	No	6d,Partial	[3]
2009	12/F	NMO	Aphasia,Delirium,Coma	0.25	Yes(U)	U	No	IVMP,IVIg	F,P,O,Corpus callosum	No	300d,Partial	[3]
2010	53/F	ON	Headache,Giddiness	U	U	U	IVMP/5d	U	P,O,T	Yes	U	[4]
2010	35/F	NMO	Sleepiness,Delirium, Visual impairment	3	Yes(30)	U (normal)	IVMP,IVIg,PLEX, Rituximab /Ud	U	T	Yes	120d,Partial	[5]
2013	U	NMO	Delirium	U	U	U (rising)	U	AH	O	U	U	[6]
2014	42/F	NMO	Limb weakness, Visual impairment	10	No	124/78	Sjogrena and Felty syndrome, Rituximab/Ud	Methotrexate	P,O	Yes	120d,Partial	[7]
2015	27/F	NMO	Headache, Visual impairment， Walking instability	3	U	176/100	PLEX/Ud	AH	P	Yes	U	[8]
2015	48/F	NMO	Epilepsy,Visual impairment	0.9	Yes(3)	U	IVMP/6d	Reducing IVMP	O,T, Centrum semiovale	Yes	U	[9]
2019	51/F	NMO	Headache	16	Yes(3)	202/127	IVMP/Ud	AH、PLEX	Brain stem	No	3d,Complete	[10]
2020	57/F	NMO	Epilepsy	4	Yes(1)	110/70–130/80	PLEX/Ud	AE	F,P,C	No	U	[11]
2020	25/F	NMO	Epilepsy	U	No	127/97	Sjogrena syndrome /Ud	IVMP	O,P,C, Brain stem, Cervical spinal cord	No	3d,Partial	[12]

*PRES* posterior reversible encephalopathy syndrome; *rLETM* recurrent longitudinally extensive transverse myelitis; *NMO* neuromyelitis optica; *ON* optic neuritis; *PLEX* plasma exchange; *IVIg* IV immunoglobulin; IVMP IV methylprednisolone; *AH* anti-hypertension; *U* unavailable; *O* Occipital lobe; *P* parietal lobe; *F* frontal lobe; *T* temporal lobe; *C* cerebellum

All cases were female, including 3 Asians and 11 Europeans, with a mean age of 42.85 years old (ranging from 12 to 57 years). All cases were diagnosed with NMOSD, and there were 2 cases of recurrent longitudinally extensive transverse myelitis (rLETM), 1 case of optic neuritis (ON), and 11 cases of NMO. The mean disease course was 7.01 years (ranging from 0.25 to 24 years). The attack interval between NMOSD and PRES was 9.83 days (ranging from 1 to 30 days). Consciousness or vision disorders occurred in more than half of the cases. Headache, epilepsy, and limb weakness were also common. It was found that 7 of the 14 patients appeared to have varying degrees of hypertension (146/86 to 220/140 mmHg). The bilateral temporal, occipital, and parietal lobes were the most common lesion locations. Of 14 patients, 10 received intravenous immunoglobulin, glucocorticoids, plasma exchange, or cytotoxic drugs, respectively. Although all the patients received symptomatic treatment (including measures to control dehydration and blood pressure), there was heterogeneity in the administration of immunotherapy treatment following the occurrence of PRES: 6 patients had enhanced or replaced immunotherapy regimens, 1 patient had reduced glucocorticoids, and other patients continued the original treatment. Significant differences existed among the cases for their regression time and degree of PRES lesions.

NMOSD is an autoimmune disease related to ON and rLETM, and is due to the action of IgGs against aquaporin-4 (AQP-4). AQP-4 is an aquaporin (AQP) ubiquitous in the central nervous system and is expressed in the perivascular end-foot of astrocytes which constitute the blood-brain barrier (BBB) together with vascular endothelial cells and astrocytes. AQP-4 plays an important role in regulating water homeostasis across the BBB [13]. AQP-4 IgGs attack the optic nerve, spinal cord, brainstem, area postrema, and cerebrum in NMOSD, according to the distribution of AQP-4 [14]. Hence, NMOSD is usually defined as AQP-4 IgG-induced immune inflammation in the central nervous system [15].

PRES is characterized by symmetrically-distributed vasogenic edema in the white matter of the bilateral frontal, parietal, occipital, temporal, and subcortical regions [16]. Asymmetries or lesions of the cerebellum, brainstem, basal ganglia, and spinal cord are rare [17]. Increased cerebrovascular exfiltration (caused by hypertension or cerebral hyperperfusion and vascular endothelial cell dysfunction) can induce PRES. Its various neurological symptoms include headache, impairment of consciousness, seizures, visual abnormalities, and focal neurological deficits.

Magana et al. speculated that vasogenic edema caused by AQP-4 IgGs in NMOSD might sometimes present as PRES [3]. Other reports have also supported this hypothesis, and one even presumed that a diagnosis of NMOSD should be considered on the basis of ON with PRES imaging [4, 5, 10]. However, PRES was also interpreted as a complication of NMOSD due to cytotoxic drug exposure, blood pressure fluctuations, etc. [11]. In conducting an analysis of the previous case reports, we found some patients’ NMOSD and PRES to have had simultaneous onsets of attack and lacked inducing factors in the prodromal stage [3, 7, 12]. Additionally, the asymmetric distribution, atypical location, and non-regression of some PRES lesions [3, 5, 7, 10, 12] all suggest that PRES may be a special manifestation of NMOSD. On the contrary, definite inducing factors and characteristic lesions support PRES as a complication of immunotherapy in the treatment of NMOSD [3, 4, 6, 9]. Thus, the reason of NMOSD concurrent with PRES is uncertain.

No standard treatment strategy has been proposed for PRES following NMOSD at present. Based on PRES, controlling a hypertension crisis and maintaining the stability of blood pressure is essential [18]. In case of seizures anti-epileptic treatment is important due to the fact that frequent seizures will worsen the brain edema [19]. Eliminating PRES-inducing factors, including AQP4-IgG and immunotherapy, will improve the prognosis in the early stage of PRES [20]. There is a question of whether NMOSD immunotherapy should be enhanced or attenuated during PRES onset,and no definite conclusion can yet be reached for it. A previous retrospective study suggested that for patients of stem cell transplantation experiencing PRES after receiving tacrolimus, there were no differences in mortality among maintaining the same dose, suspension, and replacement of tacrolimus [21]. For some immune diseases like systemic lupus erythematosus (SLE) leading to PRES, strengthening immunization is feasible [22]. Based on the above, we recommend dialectical treatment, and that should be enhanced when AQP-4 IgGs are pathogenic factors, but immunotherapy should be suspended or weakened when doubt exists on whether immunotherapy is an evoking factor. For unexplained PRES, changing or maintaining the existing immunotherapy treatment may be worth trying.

In this case, We found this woman facial swelling exacerbated significantly during the initial treatment of methylprednisolone. This might suggest that the patient’s high sensitivity for glucocorticoid,and the vascular endothelial cell dysfunction causing PRES might be more inclined to happen to her.

Overall, the etiology of PRES following NMOSD is not entirely understood. AQP-4 IgGs, unstable blood pressure, and immunotherapy might all be underlying causative factors. Unclear mechanisms lead to inconsistent treatment, and existing immunotherapy can be suspended, weakened, enhanced, replaced, or maintained according to different PRES predisposing factors.

## Data Availability

The datasets are available from the corresponding author on reasonable request.

## Abbreviations

PRES: Posterior reversible encephalopathy syndrome
NMOSD: Neuromyelitis optica spectrum disorders
NMO: Neuromyelitis opticapathogenic
AQP-4: Aquaporin-4
